# Acknowledgement to Reviewers of *Pharmaceutics* in 2018

**DOI:** 10.3390/pharmaceutics11010046

**Published:** 2019-01-21

**Authors:** Pharmaceutics Editorial Office

**Affiliations:** MDPI, St. Alban-Anlage 66, 4052 Basel, Switzerland

Rigorous peer-review is the corner-stone of high-quality academic publishing. The editorial team greatly appreciates the reviewers who contributed their knowledge and expertise to the journal’s editorial process over the past 12 months. In 2018, a total of 291 papers were published in the journal, with a median time to first decision of 15 days and a median time to publication of 37 days. Among the reviewers who reviewed for *Pharmaceutics* in 2018, the following three have been selected to receive the “*Pharmaceutics* 2018 Outstanding Reviewer Award” for the timeliness and quality of their review reports in 2018:

Wibowo, David from Griffith University, Australia
Ioannis, Nikolakakis from Aristotle University of Thessaloniki, Greece
Perinelli, Diego Romano from University of Camerino, Italy

The editors would like to express their sincere gratitude to the following reviewers for their cooperation and dedication in 2018:
Abejón, RicardoMahlin, DennyAchour, BrahimMaiolo, DanieleAdrover, AlessandraMajor, IanAhmed, MaryaMaki, YasuyukiAkamatsu, MikiMalekigorji, MaryamAkhlaghi, Seyedeh ParinazMallavia, RicardoAlam, Mohammad ParvezManca, Maria LetiziaAlder, JaneManconi, MariaAl-Fatimi, MohamedMandava, NandaAl-khattawi, AliManiruzzaman, MohammedAlkilani, Ahlam ZaidMarasini, NirmalAlmeida, HugoMarchesan, SilviaAlmoazen, HassenMarco, Iolanda DeAmoaku, Winfried M.Mareschi, KatiaAnanikov, Valentine P.Marin, José Juan GarcíaApartsin, EvgenyMarkl, DanielAppelhans, DietmarMartinez, FlemingArdejani, MaziarMartínez-Máñez, RamonAttila, BaloghMartins, NatáliaAyers, PhilipMarto, JoanaBabu, DineshMashima, RyuichiBabu, R. JayachandraMassari, SerenaBácskay, IldikóMastinu, AndreaBaek, Jong-SuepMatás, CarmenBai, ShuhuaMatikonda, SiddharthBalalaeva, IrinaMatyas, GaryBalogh, AttilaMazzaglia, AntoninoBandyopadhyay, SulalitMcardle, PatrickBansal, ArvindMcConville, ChristopherBarbalexis, PanagiotisMcHugh, KevinBardi, GiuseppeMedina-Sánchez, MarianaBarshtein, GregoryMenet, Marie ClaudeBasit, AbdulMenon, JyothiBatzias, GeorgiosMichniak-Kohn, Bozena B.Bellan, Leon M.Mignani, SergeBenson, HeatherMikhailov, Theresa ABersch, BeateMilano, FrancescoBianco, I.D.Milasius, RimvydasBidwell, III, GeneMinato, Ken-ichiroBlasi, PaoloMishra, Pawan KumarBodenstine, ThomasMiura, TakashiBölükbas, DenizMiyamoto, HirotakaBombard, SophieMlejnek, PetrBonadies, IreneMobbili, GiovannaBouropoulos, NikolaosMohammed, YousufBouyer, FredericMohankumar, KumaravelBozzani, AntonioMohanram, HariniBraatz, RichardMohtaram, Nima KhademBraga, SusanaMonroe, Mary Beth BrowningBrocks, Dion R.Montenegro, LuciaBučar, Dejan-KrešimirMonti, DanielaBurgalassi, SusiMoreau, RegisButtini, FrancescaMotohashi, HideyukiCapasso, RaffaeleMourtas, SpyridonCapriati, VitoMoyano-Mendez, Jose RamonCaputo, Gregory A.Mozafari, M. R.Caramella, Carla MMuthuramu, IlayarajaCarboni, LuciaNa, Dong HeeCaretta, AntonioNaik, ShivangiCarfagna, CosimoNair, RemyaCascone, SaraNajlah, MohammadCasettari, LucaNance, ElizabethCatanzano, OvidioNand, AshveenChan, Chun WaiNavarro, ManuelChan, K.L.Nazzal, SamiChandasana, HardikNeau, Steven H.Chandrudu, SaranyaNeves, VeraChang, Tsui-LingNgamcherdtrakul, WorapolChaudhari, Shilpa PraveenNguyen, Ba Thuy LinhChauhan, NeerajNi, DalongChen, DaiqinNicholas T., BelloChen, JunNikolakakis, IoannisChen, Kuo-YuNoriyasu, KameiChen, MingshengNosoudi, NasimCheng, Jya-WeiNounou, Mohamed IsmailCheng, Kuan-ChenNovio, FernandoCheng, XingguoNtie-Kang, FideleChetoni, PatriziaNuutila, KristoCho, Cheong-WeonObata, YasukoChoi, Hyo-JickOgawa, NorikoChoquesillo-Lazarte, DuaneOh, Jung HunChou, JoshuaOkesola, BabatundeChou, Shih-FengOliveira, Rita Manuela Palmeira DeChow, Aviva Shing FungOlson, KenChowdhury, EzharulOnishi, HirakuChu, DafengOtero-Espinar, Francisco JavierChun, Heung JaeOzaki, ToshinoriCicala, GianlucaOzsvari, BelaCiccarelli, RenataPadrela, LuisCicciù, MarcoPalamà, IlariaCiriza, JesusPalermo, EdmundCoburn, JeanninePalumbo, GiancarloCodacci-Pisanelli, GiovanniPanicker, RajeshColeman, AnthonyPanikkanvalappil, Sajanlal R.Cook, Michael T.Pantazaki, Anastasia A.Cordo Russo, Rosalia I.Panzarini, ElisaCortesi, RitaPaolo Busardó, FrancescoCostello, HeatherPapaccio, GianpaoloCourtenay, AaronPapadopoulou, LefkotheaCrucho, Carina Isabel CorreiaPapet, IsabelleCurtis, AnthonyPapini, EmanueleDa Silva-Júnior, Arnóbio AntonioParisi, FilippoDal Piaz, FabrizioPatrick, Kennerly S.Dangol, ManitaPatrinos, GeorgeDanielak, DorotaPatterson, JenniferDanielsen, Erik MichaelPavlov, Georges M.Darafsheh, ArashPedersen, HenrikDash, RanjeetPeltonen, LeenaDatta, KausikPeng, TengDavid, BerryPensabene, VirginiaDavis, MarkPerche, FédéricoDe Beer, ThomasPereira, Maria Do CarmoDe Berardis, DomenicoPerepelyuk, MarynaDe Carlo, FlaviaPerez-Stable, CarlosDe Caro, VivianaPerinelli, Diego RomanoDe La Mata, Francisco JavierPerissutti, BeatriceDe Marco, RossellaPinheiro, MarinaDe Rosa, GiuseppePinto Reis, Catarina PintoDel Pozo-Rodríguez, AnaPiosik, JacekDelgado-Calle, JesusPitterl, FlorianDeng, XiaoranPiu, SahaDeplazes, EvelynePlesner, TorbenDeshpande, ShayuPlogsted, SteveDi Donato, MarziaPopat, AmiraliDi Martino, PieraPoplawski, TomaszDi Marzio, LuisaPoudel, BarunDi, LiPoupot, RemyDing, JianxunPozo, Óscar J.Diogo, Hermínio P.Prats, Anne-CatherineDobrzynski, PiotrPruvost, AlainDong, XiaoweiPrzybyłek, MaciejDonnelly, RyanPunzo, FrancescoDrolet, BenoîtQi, ShengDu, JuanjuanRácz, AnitaDugar, Rohit P.Radchenko, Eugene V.Duval, Raphaël E.Rades, ThomasEdirisinghe, MohanRagusa, AndreaElaissari, AbdelhamidRahimi, FaridElhissi, AbdelbaryRai, PrakashElnahhas, ToqaRaimi-Abraham, BahijjaEl-Seedi, Hesham R.Raimondi, LaviniaElShaer, AmrRais, RanaEngelke, HannaRajendran, Jagathesh ChandraEsposito, GennaroRamasamy, MohankandhasamyEspuelas, SocorroRamirez-Vick, JaimeEstelrich, JoanRavaioli, StefanoEsteve-Romero, JosepRavula, AbhigyanEugster, PhilippeRen, KaixuanFábián, LászlóReyes-Garcés, NathalyFalco, AlbertoRice, Peter J.Fang, Hsu-WeiRichardson, JosephFardel, OlivierRichardson, SimonFasinu, Pius S.Rinaldi, FedericaFatouro, DimitrisRistori, SandraFelgueiras, HelenaRizzolio, FlavioFelix, Klaus M.Roberts, MattFeng, XinRodríguez-Hornedo, NaírFenyvesi, EvaRom, SlavaFerenz, KatjaRomero-Lorca, AliciaField, Martha S.Ronain Smith, BryanFilipovic, MilosRosado, CatarinaFiorino, FerdinandoRosania, GusFirlak, MelikeRosenholm, JessicaFisher, JeffreyRosière, RémiFlament, Marie-PierreRuiz Rubio, LeireFlorczyk, Stephen J.Russo, AnnapinaFloresta, GiuseppeSaenz Del Burgo, LauraFonteh, AlfredSainlos, MatthieuFreire, JoaoSalameh, Therese S.Fuhrmann, GregorSalvalaglio, MatteoFukuta, TatsuyaSamanta, SubarnaFuller, AmeliaSánchez-García, DavidFutakuchi, MitsuruSangaraju, DewakarGadde, SureshSani, GabrieleGangloff, SophieSanjuán, Virginia MerinoGao, HuileSansone, FrancescaGarbayo, ElisaSanti, PatriziaGaspar, DianaSantos De Almeida, TâniaGhica, Mihaela-VioletaSara, ZalbaGiacomelli, ChiaraSarantopoulou, EvangeliaGiannoni, PatriziaSarma, SauravGigoux, VéroniqueSarmento, BrunoGikas, EuaggelosSato, HideyukiGiovannelli, PiaSavary, StéphaneGloria, AntonioSaville, DorothyGok, OzgulScala, AngelaGomes, Maria S.Schiavone, MarcoGonçalves, Lidia M DSchneider, FelixGong, HuaSchnichels, SvenGoreham, ReneeSchönthal, Axel H.Gosselet, FabienSchumann, CananGrisoni, FrancescaSchwaminger, SebastianGrossi, GiancarloSękowski, SzymonGugliandolo, EnricoSen Karaman, DidemGuillaume, OlivierSerda, RitaGunda, SriramSerpico, RosarioGupta, MohitSerrano, Dolores R.Gura, KathleenSeverino, BeatriceHagenbuch, BrunoSeyfoddin, AliHama, SusumuShah, ManishHamad-Schifferli, KimberlyShamay, YosefHandzlik, JadwigaSharma, AjitHao, JinsongShavandi, AminHaque, TasnuvaShcharbin, DzmitryHardy, John G.Shi, YiHare, ColinShimada, TakashiHaware, Rahul V.Shimpi, Manishkumar R.Hayakawa, TohruShityakov, SergeyHays, FranklinSignore, GiovanniHe, HongliangSilva, Margarida F. B.Hernan, AmandaSimitzi, CharaHerwadkar, AnushreeSimões, SandraHigaki, KazutakaSimone, ElenaHinck, AndrewSimon-Yarza, TeresaHinrichs, Wouter L.J.Simpson, Garth J.Hirayama, FumitoshiSimpson, Julie E.Hofmann, UteSingh, ArunimaHopkins, SamanthaSinha, AbhijeetHorhat, Florin GeorgeSiwale, RodneyHoskins, ClareSkiba, MohamedHossain, Md EkhtearSloan, Kenneth B.Hsu, Fu-YinSłoczyńska, KarolinaIatridi, ZacharoulaSmiesko, MartinIatrou, HermisSobeh, MansourIerapetritou, MarianthiSolanki, NayanIleana Ionescu, MihaelaSoleymani Abyaneh, HodaIndiveri, CesareSolinís, M.ÁngelesIno, KosukeSomani, SukrutInoue, YutakaSonvico, FabioIoannis, NikolakakisSorrell, J. MichaelIshii, YujiSorrenti, MilenaIslam, Muhammad TariqulSottnik, JosephIta, KevinSpanakis, MariosIto, TaichiSpingler, BernhardIwasaki, TakashiSriram, GopuJain, AkshayStagni, GraziaJaiswal, JagdishŠtěpánek, PetrJamwal, RohitashStewart, Michael W.Jaradat, NidalStieger, BrunoJaumot, JoaquimSumi, ShoichiroJayant, Rahul DevSun, ChenJelokhani-Niaraki, MasoudSun, MengJensen, Katrine Birgitte TarpSun, WujinJeong, Hyun YoungSun, YuanJicsinszky, LászlóSurber, ChristianJin, JiefuSutaria, DhruvitkumarJindrich, JindrichSzweda, PiotrJones, JeremyTabata, YasuhikoJuang, Shin-HunTaghibiglou, ChangizJug, MarioTampucci, SilviaJung, HyungilTaratula, OlenaJung, JaehanTavares, AnthonyJung, JuyeonTaylor, Erin AudreyJurado, Amalia S.Telukutla, Srinivasa ReddyK A Foyez, MahmudThakur, SachinKachrimanis, KyriakosThamaraparambil Jayaram, DhanyaKaczor, Agnieszka A.Thommes, MarkusKalia, YogeshvarTian, JustinKambhampati, Siva PramodhTimin, Alexander S.Kanaujia, ParijatTing, Richard C. H.Karcz, DariuszTomaiuolo, GiovannaKarolewicz, BozenaTomalia, Donald A.Katayama, YoshikiTong, ShengKathawala, RishilTouitou, ElkaKathuria, HimanshuTownley, Helen E.Katsumi, HidemasaTrotta, FrancescoKauppinen, AnuTsai, Shiao-WenKawakami, KohsakuTseng, Ching-LiKello, MartinTsiourvas, DimitrisKerr, IanTsugawa, HiroshiKevadiya, BhaveahUekusa, HidehiroKevadiya, BhaveshUeng, Yune-FangKharkar, PrathameshVaidya, AmitaKhisamutdinov, EmilVaidya, BhuvaneshwarKhutoryanskiy, VitaliyVan Bocxlaer, KatrienKim, BohyungVandooren, JenniferKim, Seong-GonVanić, ŽeljkaKim, TaisunVankayala, RavirajKlajnert-Maculewicz, BarbaraVarga, ZoltanKlar, AgnesVasic, VesnaKoetz, JoachimVautier, SimonKoga, TomoyukiVenkataraman, ShrinivasKöhler, KarstenVenturini, CristinaKolluru, GopiVial, LaurentKootala, SujitVillares, Jose Manuel MorenoKopel, PavelVllasaliu, DritonKotsuchibashi, YoheiVrabič Brodnjak, UrškaKotta-Loizou, IolyVuddanda, Parameswara RaoKoutelidakis, Antonios E.Wagemaker, Tais Aleriana LuconKratz, HaraldWaldmeier, FelixKrupa, AnnaWalker, GregKuang, HuihuiWalker, HeatherKucuk, IsrafilWang, Jian-WenKumar Patel, SravanWang, LiliKumar, PraveenWang, PengchengKurcok, PiotrWang, WeiKurrikoff, KaidoWang, XiaofengKwade, ArnoWang, YifeiLaforenza, UmbertoWang, YingLalatsa, AikateriniWang, ZhijunLallana, EnriqueWarnken, ZacharyLamberti, GaetanoWeng, Ching-FengLamprou, DimitriosWesołowska, OlgaLandreville, SolangeWhitehead, Debra E.Larraneta Landa, EnekoWibowo, DavidLauro, Maria RosariaWin, Khin YinLayek, BuddhadevWinkler, HeinzLee, Chia-HungWitkowska Nery, EmiliaLee, Jae-HoWong, Clarence T. T.Lee, JaehwiWright, DavidLee, SangkilWu, ErxiLee, Tae-RinWu, Yu-TseLee, TuWuttke, StefanLeiviskä, KaukoXia, TianLeong, Thomas Seak HouXu, ChunLeporatti, StefanoXu, RaymondLeung, IvanhoeXu, TaoLexchin, JoelXu, Zhi Ping (Gordon)Li, MengYallapu, MuraliLi, NaYamada, HiroyukiLi, S. KevinYang, GuangLi, WenyiYang, Sherry X.Li, YanYang, Yueh-Hsun KevinLiao, Ai-HoYannakopoulou, KonstantinaLin, Hai-ShuYanni, SouzanLin, RanYAU, WaiLin, Wei-ChihYoon, In-SooLin, ZhoumengYoon, OkjaLinder, StigYoung, SimonLiu, Cheuk LunYousefi, Azizeh-MitraLiu, FangYu, ZaiqunLiu, HuolongYuba, EijiLiu, XifengZafar, MuhammadLlacua, L. AlbertoZaidi, SaheemLocatelli, MarcelloZarzycki, Pawel K.Lochhead, Jeffrey J.Zeglinski, JacekLöhr, MatthiasZhang, WujieLu, MingZhang, YuLuan, YuxiaZhao, ZongminLucarini, SimoneZhu, QiuyuLuk, FrederickZhu, WeiLuo, RongcongZiaee, AhmadMa, YongZimmermann, KatharinaMaeng, Han-JooZloh, MireMagnani, MauroZuvela, PetarMaher, Sam


